# Application of impulse oscillometry to detect interstitial lung disease and airway disease in adults with rheumatoid arthritis

**DOI:** 10.1186/s12890-023-02615-0

**Published:** 2023-09-08

**Authors:** Wen-Chien Cheng, Shih-Hsin Chang, Wei-Chun Chen, Bing-Ru Wu, Chia-Hung Chen, Chi-Chien Lin, Wu-Huei Hsu, Joung-Liang Lan, Der-Yuan Chen

**Affiliations:** 1 Division of Pulmonary and Critical Care, Department of Internal Medicine, China Medical University Hospital, Taichung, Taiwan; 2 Department of Life Science, National Chung Hsing University, Taichung, Taiwan; 3 Ph.D. Program in Translational Medicine, National Chung Hsing University, Taichung, Taiwan; 4 Rong Hsing Research Center for Translational Medicine, National Chung Hsing University, Taichung, Taiwan; 5 Rheumatology and Immunology Center, China Medical University Hospital, Taichung, Taiwan; 6 and Animal Biotechnology Center, Institute of Biomedical Science, the iEGG , National Chung-Hsing University, Taichung, Taiwan; 7 College of Medicine, China Medical University, Taichung, Taiwan; 8 College of Medicine, National Chung Hsing University, Taichung, Taiwan

**Keywords:** Rheumatoid arthritis, Interstitial lung disease, Small airway disease, Impulse Oscillometry

## Abstract

**Background:**

We conducted a retrospective observational study to explore the potential application of impulse oscillometry (IOS) as an alternative to high-resolution computed tomography (HRCT) for detecting pulmonary involvement in patients with rheumatoid arthritis (RA) because clinically evident interstitial lung disease (ILD) and airway involvement are common in this population.

**Methods:**

We enrolled 72 patients with RA who underwent pulmonary function tests (PFTs) and IOS between September 2021 and September 2022. We aimed to identify the PFT and IOS variables associated with lung diseases shown on HRCT images.

**Results:**

In our cohort of 72 patients, 48 underwent HRCT; of these, 35 had airway disease or ILD and 13 showed no obvious abnormalities on HRCT. Abnormal IOS and PFT parameters were observed in 34 and 23 patients, respectively, with abnormal HRCT images. The predicted percentages for forced vital capacity, the ratio of forced expiratory volume in the first one second to forced vital capacity, and forced mid-expiratory flow value were significantly lower in patients with abnormal HRCT. Lung resistance at 5 Hz, difference in resistance between 5 and 20 Hz, resonant frequency (Fres), and reactance area were higher in these patients and reactance at 5 Hz was lower. Compared to other parameters, Fres > 14.14 was significantly associated with alterations in HRCT and may be used as an indicator for monitoring disease.

**Conclusion:**

Fres > 14.14 is significantly associated with lung involvement in RA patients. Performance of spirometry with IOS is more beneficial than spirometry alone for evaluating lung involvement in RA patients.

**Supplementary Information:**

The online version contains supplementary material available at 10.1186/s12890-023-02615-0.

## Introduction

Rheumatoid arthritis (RA) is a systemic chronic inflammatory disease that leads to chronic synovitis, joint destruction, and poor life quality [1]. Up to 80% of RA patients have one or more comorbidities [2, 3] resulting in the shortening of their life span [4]. Pulmonary diseases are prevalent among 60–80% of RA patients and are significant because they lead to a mortality rate of 10–20% [5–7]. RA can involve the lungs in various ways and cause diseases of the parenchyma, airways or pleura, e.g., interstitial lung disease (ILD), small airway disease (SAD), and pleural disease, but less commonly of pulmonary vessels [8]. High resolution computed tomography (HRCT) of the chest, along with pulmonary function tests (PFTs), are widely employed for identifying pulmonary abnormalities in RA patients [9]. However, frequent HRCT imaging can result in a substantial amount of radiation exposure. Spirometry may have limitations in detecting abnormalities in the small airways, and it may be difficult to obtain reliable results from patients with severe lung dysfunction who are unable to perform the necessary maneuvers. Optimal screening, diagnostic, and treatment strategies for RA-associated pulmonary diseases are still lacking and are the focus of current research.

Spirometry is a PFT that measures air volume displacement during forced respiratory maneuvers. In contrast, impulse oscillometry (IOS) does not depend on the patient's effort. It evaluates changes in airway pressure and flow and translates them into respiratory system resistance (Rrs) and reactance (Xrs). A significant benefit of IOS is that it can be measured during regular breathing [10]. Rrs is an indicator of airway caliber and Xrs is believed to represent the elastic and inertial characteristics of the respiratory system. The use of IOS has significantly gained in popularity for assessment of disease status and therapeutic efficacy in obstructive pulmonary conditions, such as chronic obstructive pulmonary disease and bronchial asthma [11–13]. IOS is also useful in assessing ILD [14–16]. Multiple investigations have validated its clinical utility. Therefore, IOS has potential as an application to identify RA patients’ respiratory abnormalities, which cannot be detected through spirometric examinations.

Studies of complete assessment of RA-related pulmonary pathologies with the use of IOS are currently lacking. Sokai et al. suggested that the forced oscillation technique (FOT) might be a useful tool for evaluating alterations in respiratory functions in RA patients [17]. The primary distinction between FOT and IOS is that the former sequentially transmits sound waves of varying frequencies, while the latter transmits an impulse that can be decomposed into different frequencies, which reduces testing time and improves signal-to-noise resolution [10]. The objective of this pilot study was to investigate the presence of pulmonary involvement in patients with RA using two different methods—spirometry and IOS. The findings were then cross-referenced with the results obtained from HRCT scans of the chest.

## Materials and methods

### Patient enrollment

**Fig. 1:**
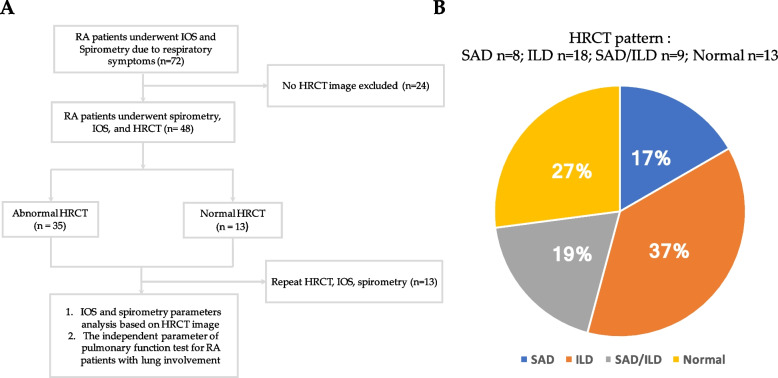
**A** Flowchart of patient enrollment in the study. **B** Various imaging patterns on HRCT scans in our cohort. HRCT: High-resolution computed tomography; ILD: interstitial lung disease; PPFE: Pleuroparenchymal fibroelastosis; SAD: small airway disease

Seventy-two patients of the China Medical University Hospital who met the 2010 revised criteria of the American College of Rheumatology for RA [18] were enrolled in this study between September 2021 and September 2022. Of these patients, 48 underwent tests including IOS, spirometry, and HRCT. We divided these 48 patients into two groups: the first group consisted of patients with abnormal findings on their HRCT scans, while the second group consisted of patients with normal findings. We established relationships between measurements and parameters from pulmonary function tests (IOS and spirometry) and chest CT images and identified the independent parameter of pulmonary function for RA patients with lung involvement (Fig. 1A). Clinical data, including patient history and laboratory findings, were obtained from medical records. On the same day of examination, respiratory impedance was measured using IOS and pulmonary function tests were performed. HRCT images taken within three months before or after the examination day were reviewed.

### HRCT images

The appearance of the lungs, including the presence of SAD and ILD, was evaluated using HRCT scans obtained with a GE Optima 660 128 slice CT scanner (technical scan parameters: 100–390 mA and 120 kV). The images were displayed at window and level settings optimized for the visualization of lung parenchyma (level: − 800 HU; width: 1800 HU). The interval was 2.5 mm. All digital images were reconstructed using a high-resolution algorithm and obtained at the window level appropriate for lung parenchyma and the mediastinum.

### IOS measurements

The MasterLab IOS System (Erich Jaeger, Würzburg, Germany) was used to collect IOS parameters before conventional spirometry parameters. Calibration was performed using a single volume of air (3 L) at different flow rates and a reference resistance device (0.2 kPa/L/s). Patients wore a nose clip and a manufacturer-provided hard plastic oval mouthpiece to minimize leakage of expired air. Cheek support was instructed to decrease shunt compliance of the cheeks. Artifacts due to coughing, breath holding, swallowing, or vocalization were excluded. All IOS measurements were performed by a single, experienced respiratory technician. The parameters assessed included resonant frequency (Fres), resistance at 5 Hz (R5), resistance at 20 Hz (R20), the difference between resistance at 5 and 20 Hz (R5-R20), reactance at 5 Hz (X5), area of reactance (AX), and impedance at 5 Hz (Z5). Abnormal values of IOS parameters were defined as any of the following: R5 > 150%; R20 > 150%; R5-R20 > 0.07; Fres > 14.14; X5 < -0.12; and AX > 0.44 [19].

### Pulmonary function test

Spirometry was performed five minutes after IOS measurements using a pneumotachometer system equipped with a Lilly head (MasterScreen system; Erich Jaeger). The spirometric flow–volume curve was determined according to international criteria [20]. Patients used a nose clip and were instructed on how to perform a standard forced expiratory maneuver. Each data set included at least three reproducible attempts, with a maximum of eight attempts made by each patient. The best result from the three attempts was selected for the final data analysis. Lung function measurement was repeated 20 min after administration of three puffs of 100 μg of albuterol via a pressurized metered dose inhaler. The evaluated parameters were forced vital capacity (FVC), forced expiratory volume in one second (FEV1), maximum mid-expiratory flow between 25 and 75% of the forced vital capacity (MMEF25-75), inspiratory capacity (IC), total lung capacity (TLC), functional residual capacity (FRC), residual volume (RV), and diffusing capacity for carbon monoxide (DLco). Abnormal values of PFT parameters were defined as any of the following: FVC < 80% of predicted value; FEV1 < 80% of predicted value; MMEF25-75 < 65% of predicted value; RV > 140% or < 80% of predicted value; DLco < 75% of predicted value; and TLC > 120% or < 80% of predicted value [21].

### Determination of serum levels of rheumatoid factor-immunoglobulin (Ig)M and anti-cyclic citrullinated peptide antibody IgG levels

Serum levels of rheumatoid factor-immunoglobulin (Ig)M were determined using IMMAGE® Immunochemistry Systems and Calibrator 5 Plus (Beckman Coulter Ireland Inc., Mervue Business Park, Mervue, Galway, Ireland). Levels < 20 IU/mL were considered negative. The anti-cyclic citrullinated peptide (anti-CCP) antibody IgG levels were determined using the EliA™ technique (POhadia 250; Thermo Fisher Scientific, Uppsala, Sweden). Levels were considered negative if they were < 7 U/mL, equivocal if 7–10 U/mL, and positive if > 10 U/mL.

### Statistical analysis

This study used the independent two-sample *t*-test to compare continuous variables, which were expressed as mean ± standard deviation. Categorical variables were expressed as numbers and percentages and compared using either the χ2 test or Fisher's exact test. To account for baseline differences between asthmatic and non-asthmatic patients, propensity score matching (1:1) was performed using age, sex, height, and BMI as covariates. Multiple logistic regression was performed to identify factors associated with the composite outcome. In addition, the area under the curve (AUC) and its 95% confidence interval (CI) were calculated using the nonparametric method. Cutoff values were determined using Youden's index. Statistical significance was established at *p*-values < 0.05, and 95% CIs were calculated. Statistical analyses were performed using MedCalc for Windows version 18.10 (MedCalc Software, Ostend, Belgium).

## Results

### Clinical characteristics of RA patients

We stratified the patients into two groups based on the presence or absence of abnormal findings on their HRCT scans. As shown in Table 1, elderly patients with high levels of serum anti-CCP and CRP were more likely to have abnormal HRCT images. Patients with pulmonary involvement had significantly lower FVC%, FEV1/FVC%, and MMEF25-75% values, compared to those without lung involvement shown on HRCT. In addition, patients with abnormal HRCT scans had higher AX, Fres, R5, and R5-R20 and lower X5 values compared to patients with normal HRCT scans.

**Table 1:** Baseline clinical characteristics and differences in spirometry and IOS parameters between patients with rheumatoid arthritis with normal and those with abnormal findings in HRCT

	**Abnormal on HRCT (*n* = 35)**	**Normal on HRCT (*n* = 13)**	***p*-value**
Age, years	69.0 (65.0–70.0)	55.0 (44.2–60.9)	< 0.001
Sex, female (%)	28 (80.0)	10 (76.9)	0.818
RF	234.4 (101.5–367.2)	129.5 (38.7–220.4)	0.183
RF( +) (%)	19 (54.3)	8 (61.5)	0.656
Anti-CCP	381.2 (198.6–563.7)	114.5 (6.67–222.3)	0.013
Anti-CCP( +) (%)	24 (68.6)	9 (69.2)	0.965
CRP	0.56 (0.23–0.89)	0.18 (0.10–0.25)	0.029
ESR	18.8 (13.1–24.5)	17.5 (7.68–27.4)	0.814
Spirometry			
FVC (%)	91.9 (87.3–95.7)	102.4 (95.6–110.8)	0.038
FEV1/FVC (%)	79 (75.2–81.0)	82 (79.1–86.0)	0.024
MMEF25/75 (%)	57.6 (48.9–66.1)	77.9 (71.9–102.5)	0.001
RV (%)	102 (91.6–109.4)	106.0 (89.6–118.6)	0.492
IOS			
R5 (%)	150 (128.7–176.3)	110 (93.6–128.0)	0.004
R20 (%)	132 (118.3–146.7)	116 (106.0–133.8)	0.107
X5 < -0.12	-0.18 (-0.22–-0.15)	-0.13 (-0.15–-0.11)	0.011
R5-R20 > 0.07	0.13 (0.06–0.18)	0.05 (0.01–0.08)	0.008
Fres > 14.14	16.0 (14.9–21.8)	12.1 (10.1–14.2)	0.006
AX > 0.44	1.00 (0.56–1.89)	0.40 (0.23–0.60)	0.001
DLCO (%)	66.7 (56.3–76.7)	71.3 (61.1–89.8)	0.167

*CCP* Cyclic citrullinated peptide antibody, C-reactive protein, *DLCO* Diffusing capacity of the lung for carbon monoxide, *ESR* Erythrocyte sedimentation rate, *FVC* Forced vital capacity, *FEV1* Forced expiratory volume in one second, *HRCT* High-resolution computed tomography, *IOS* Impulse oscillometry, *MMEF* Maximal mid expiratory flow curve, *RF* Rheumatoid factor, *RV* Residual volume

### HRCT findings

The ILD group had characteristic CT findings, such as ground glass opacity, reticulation, honeycombing, and traction bronchiectasis, typically associated with ILD. In contrast, the SAD group exhibited characteristic CT findings of the disease, including mosaic attenuation, air trapping, centrilobular micronodules, bronchiectasis/bronchiolectasis, and bronchiolar abnormality. In our study, SAD was used to characterize CT scans of eight patients, while ILD was used to characterize CT scans of 18 patients. Nine patients presented with both SAD and ILD, and the remaining 13 patients had normal CT scans. Pleuroparenchymal fibroelastosis (PPFE) is a rare form of ILD characterized by dense fibrosis involving the visceral pleura and the underlying subpleural parenchyma, with a particular tendency to affect the upper lobes of the lungs [22]. The presence of PPFE can coincide with connective tissue disease (CTD)-associated ILD, such as RA. A study suggested that the occurrence of PPFE in RA-ILD was 6.5% [23]. In this study, the prevalence of PPFE on HRCT was approximately 8.3% (4/48) (Fig. 1B).

### Results of PFTs and IOS

Of the 72 RA patients presenting with respiratory symptoms, 60 (83.3%) individuals’ IOS parameters were abnormal, whereas only 35 (48.6%) individuals’ PFT parameters were abnormal (Table 2). Among 35 RA patients who had abnormal HRCT images, 34 (97.1%) also had abnormal IOS parameters, while only 23 (65.7%) had abnormal PFT parameters (Table 3). We also established relationships between these pulmonary function measurements (IOS and spirometry) and chest CT images (Table supplement 1) and identified the independent parameter of the pulmonary function test for RA patients with lung involvement (Fig. 1A).

**Table 2:** Comparison between patients with rheumatoid arthritis with normal and those with abnormal spirometry and IOS parameters; all patients had respiratory symptoms. (*n* = 72)

	IOS		*p*-value
Spirometry	Normal	Abnormal	**0.002**
Normal	11	26	37 (51.4)
Abnormal	1	34	35 (48.6)
	12 (16.7)	60 (83.3)	72

*IOS* Impulse oscillometry

**Table 3:** Comparison between patients with rheumatoid arthritis with normal and those with abnormal spirometry and IOS parameters; all patients had abnormal findings on chest HRCT (*n* = 35)

	IOS		*p*-value
Spirometry	Normal	Abnormal	0.470
Normal	0	12	12 (34.3)
Abnormal	1	22	23 (65.7)
	1 (2.9)	34 (97.1)	35

*HRCT* High-resolution computed tomography, *IOS* Impulse oscillometry

### Multiple logistic regression and receiver operating characteristic analyses

We performed univariate and multivariate logistic regression analyses, one model including spirometry parameters and another including IOS parameters. The model with spirometry parameters showed a significant association between MMEF < 65% and having an abnormal HRCT image (*p* = 0.012) in univariate analysis. The model with IOS parameters showed a significant association between R5 > 150% (*p* = 0.013), AX > 0.44 (*p* = 0.006), and Fres > 14.14 (*p* = 0.003) and having an abnormal HRCT image in univariate analysis. Final models after multivariate analysis showed that Fres > 14.14 was the only selected parameter significantly associated with having an abnormal HRCT image (*p* = 0.039) (Table 4).

**Table 4:** Univariate and multivariate analyses of spirometry and IOS parameters associated with patients with rheumatoid arthritis with abnormal findings in HRCT

	**Univariate**			**Multivariate model 1**			**Multivariate model 2**		
	**HR**	**95% CI**	***p*-value**	**HR**	**95% CI**	***p*-value**	**HR**	**95% CI**	***p*-value**
**Age > 65 years**	20.31	2.36–174.7	0.006	44.04	2.65–732.1	0.008	13.41	1.47–121.8	0.021
**Anti-CCP**	1.002	0.99–1.01	0.124						
**CRP**	6.172	0.32–118.5	0.227						
**R5 > 150%**	1.031	1.01–1.06	0.013						
**R20 > 150%**	1.023	0.99–1.05	0.083						
**R5-R20 > 0.07**	2.671	0.69–10.33	0.154	0.126	0.008–1.87	0.132			
**AX > 0.44**	7.466	1.79–30.99	0.006	3.348	0.267–41.98	0.349			
**Fres > 14.14**	9.629	2.33–43.01	0.003	18.01	1.15–282.7	0.039			
**X5 < -0.12**	1.450	0.30–6.91	0.641						
**FVC < 80%**	539E + 006	-	0.997						
**FEV1/FVC < 70%**	2.001	0.211–18.95	0.545						
**MMEF < 65%**	8.250	1.58–43.02	0.012				4.421	0.736–26.53	0.104
**DLCO < 76%**	1.385	0.381–5.034	0.621						

*CCP* Cyclic citrullinated peptide antibody, C-reactive protein, *DLCO* Diffusing capacity of the lung for carbon monoxide, *FVC *Forced vital capacity, *FEV1* Forced expiratory volume in one second, *HRCT* High-resolution computed tomography, *IOS* Impulse oscillometry, *MMEF* Maximal mid expiratory flow curve

**Fig. 2:**
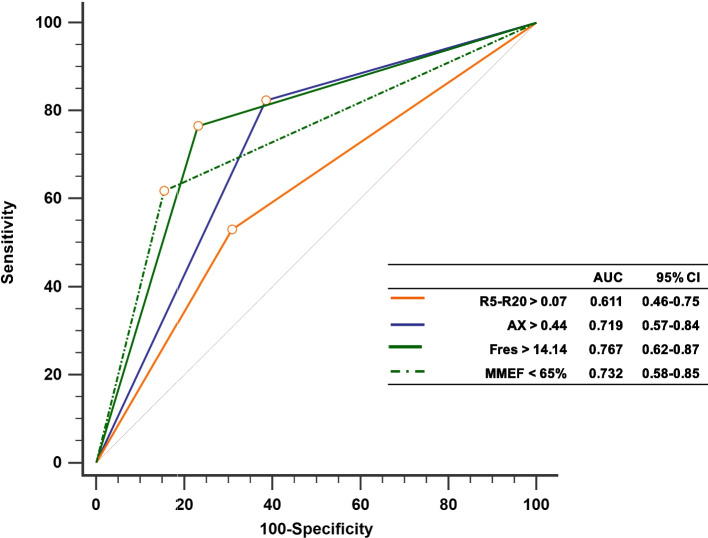
ROC curve analysis of spirometry and IOS parameters for predicting patients with abnormal findings in HRCT images. ROC: receiver operating characteristic curve; HRCT: high-resolution computed tomography; IOS: impulse oscillometry R5-R20 > 0.07 vs. AX > 0.44, *p* = 0.066; R5-R20 > 0.07 vs. Fres > 14.14, *p* = 0.011; AX > 0.44 vs. Fres > 14.14, *p* = 0.427; MMEF < 65% vs. AX > 0.44, *p* = 0.901; MMEF < 65% vs. Fres > 14.14, *p* = 0.675; MMEF < 65% vs. R5-R20 > 0.07, *p* = 0.207; R5-R20 > 0.07, Sen: 54.2% and Spe: 69.2%, *p* = 0.137; Fres > 14.14, Sen: 74.3% and Spe: 76.9%, *p* < 0.001; AX > 0.44, Sen: 82.3% and Spe: 61.5%, *p* = 0.005; MMEF < 65%, Sen: 60.0% and Spe: 84.6%, *p* = 0.009

Receiver operating characteristic (ROC) curves were generated using predictive scoring equations derived from the results of the univariate logistic regression analyses. The AUCs for R5-R20 > 0.7, AX > 0.44, Fres > 14.14, and MMEF < 65% were 0.611, 0.719, 0.767, and 0.732, respectively. The AUC value did not differ significantly between the selected spirometry and IOS parameters. However, a significant difference was observed in the AUC value between Fres > 14.14 and R5-R20 > 0.7 (*p* = 0.011) (Fig. 2).

### Changes in Fres values in longitudinal follow-up

**Fig. 3:**
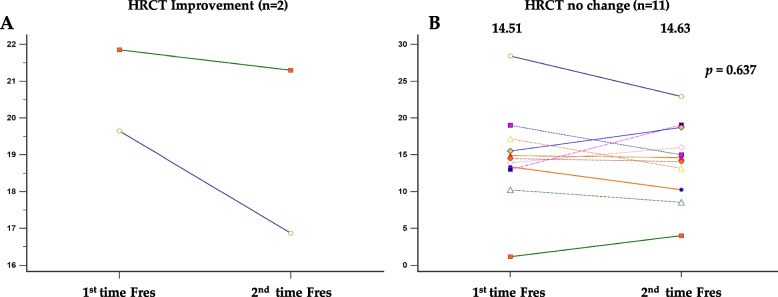
The values of Fres before and after treatment based on whether or not improvement is shown on HRCT. **A** Improvement. **B** No improvement

Thirteen patients underwent a second follow-up HRCT scan and IOS. Two patients showed a significant improvement in their HRCT findings, with a concomitant decrease in their Fres values. In contrast, the remaining 11 patients did not show any significant change in their HRCT findings, and their Fres values before and after the follow-up period also did not differ significantly (14.51 vs 14.63; *p* = 0.637) (Fig. 3).

## Discussion

To the best of our knowledge, this study is the first to use IOS parameters to evaluate pulmonary involvement in RA patients, with further assessment based on HRCT findings. The main result of this study is that IOS is more sensitive than spirometry in detecting pulmonary involvement in patients with RA. Most IOS parameters showed a significant correlation with spirometry parameters in RA patients with abnormal HRCT findings. The AUC of Fres was higher at the cutoff value of > 14.14, with higher sensitivity and specificity than other PFT parameters for predicting RA with pulmonary involvement. Therefore, Fres was not only significantly associated with pulmonary involvement in these patients, but also a potentially important indicator that can be used as a predictor before and after treatment.

In previous studies, ILD prevalence was 10% to 42% and SAD prevalence was 8% to 65% in RA patients; prevalence varied based on the detection method and patient selection criteria in each publication [24, 25]. Paulin et al. found that a significant percentage (72.2%, 60/83) of early RA patients had abnormal results on HRCT scans. In addition, patients with lung involvement had notably lower FVC% and DLCO% values, as well as higher RV/TLC values [26]. Pulmonary function test parameters, such as %DLCO, FEV1/FVC, and %FEF25-75, have been used for assessing functional impairment in pulmonary manifestations linked to RA [9, 25]. Our study also found a similar prevalence (72.9%, 35/48) of pulmonary involvement shown on HRCT images. In our study, patients with lung involvement had significantly lower FVC%, FEV1/FVC, and MMEF25-75% values. In addition, our evaluation using IOS showed that patients with pulmonary involvement had significantly higher R5, R5-R20, AX, and Fres, as well as lower X5. We also demonstrated a significant correlation between IOS parameters and both pulmonary functions and CT findings in patients affected by pulmonary diseases associated with RA. IOS seems to be able to detect more RA patients with lung involvement compared to spirometry.

Sokai et al. also showed that impedance measured by the FOT reflects abnormalities in pulmonary functions and structures in patients with RA and indicated that patients who presented with architectural distortion had a significantly higher ΔX4 value compared to those who did not exhibit such a condition [17]. In contrast, our results show that a Fres value higher than 14.14 is an independent predictor of pulmonary diseases in RA patients, similar to the finding that Fres is higher in individuals with asthma compared to those who are healthy [13, 27]. Singh et al. reported that one-third of individuals with RA had SAD, even when disease duration was short, disease activity was low to moderate, and there were no respiratory symptoms [28]. As a result, IOS is capable of identifying airway changes that do not have an impact on airflow, making it a valuable tool for assessing pulmonary involvement in RA patients, particularly in those with normal spirometry results.

IOS can comprehensively analyze diseases of the respiratory system, as well as use Rrs to analyze obstructive lung diseases and Xrs to analyze ILD. Assessment of ILD pathophysiology is believed to be more important than evaluation of Xrs. The value of AX, which represents the area under the curve of reactance from 5 Hz to Fres, can be used to evaluate the lung's elastic properties [10]. The AUC for predicting pulmonary disease in RA patients using AX > 0.44 and Fres > 14.14 were 0.719 and 0.767, respectively. Fujii et al. found that in ILD, the Fres of the inspiratory phase was correlated with FVC, FEV1, DLCO, and fibrosis score [14]. Sugiyama et al. reported that the presence of ILD was linked to ΔX5, and that there was a negative correlation between ΔX5 and VC and DLco [15]. A study of the efficacy of the FOT for evaluating idiopathic pulmonary fibrosis (IPF) disease status showed that X5 during the inspiratory phase predicted the progression of lung capacity deterioration [16]. In addition to predicting ILD in RA patients, the present study also shows that Fres may be used to monitor disease status because of its correlation with changes on chest HRCT.

Our study has several strengths: we enrolled RA patients with respiratory symptoms and we performed both HRCT and lung functional tests (spirometry and IOS), as well as laboratory investigations. Our findings suggest that early lung screening using IOS can identify early-stage abnormalities in a significant proportion of patients, thus offering the possibility of early intervention when necessary.

Despite this pilot study’s novel findings, it has some limitations. First, it was a cross-sectional observational study with a small sample size. Second, not all patients underwent HRCT in the follow-up period. Therefore, we need more patients to confirm whether the Fres could be used as an important parameter for tracking. Third, since IOS is not as widely used as spirometry, there is currently no consensus on reference values. The values of IOS have been found to be influenced by factors such as age, gender, height, body weight, BMI, and ethnicity, the cutoff values for IOS parameters suggested in this study were based on many reference values associated with adult IOS [29–31]. However, despite these limitations, the results are still clinically significant as they demonstrate the usefulness of IOS in diagnosing and monitoring pulmonary involvement in patients with RA.

## Conclusion

In summary, our study shows that a significant percentage of diagnosed RA patients have abnormal HRCT findings, with clinical respiratory symptoms. The combined use of spirometry and IOS may have superior clinical utility for diagnosing lung involvement in RA patients. IOS appears to be a more convenient procedure for patients and has higher sensitivity compared to spirometry. Among the IOS parameters, Fres has a significant association with pulmonary involvement in individuals with RA.

### Supplementary Information

**Additional file 1.**

## Data Availability

Upon reasonable request, the corresponding author is willing to provide the datasets that were used and/or analyzed in the current study.

## Abbreviations

DLco: Diffusing capacity for carbon monoxide
MMEF25-75: Maximum mid-expiratory flow between 25 and 75% of the forced vital capacity
FEV1: Forced expiratory volume in one second
FRC: Functional residual capacity
FVC: Forced vital capacity
HRCT: High-resolution computed tomography
IC: Inspiratory capacity
ILD: Interstitial lung disease
IOS: Impulse oscillometry
PFT: Pulmonary function tests
PPFE: Pleuroparenchymal fibroelastosis
RA: Rheumatoid arthritis
ROC: Receiver operating characteristic curve
RV: Residual volume
SAD: Small airway disease
TLC: Total lung capacity
