# Supplementary material for: Application of impulse oscillometry to detect interstitial lung disease and airway disease in adults with rheumatoid arthritis
Source: BMC Pulm Med. 2023 Sep 8;23:331. doi: 10.1186/s12890-023-02615-0 (PMC10485984; doi:10.1186/s12890-023-02615-0)
Supplement: Supplementary file 1 — Additional file 1. [file 12890_2023_2615_MOESM1_ESM.docx]

Table supplement 1. The relationships between these pulmonary function measurements and chest CT images

|  | **ILD on HRCT(n=18)** | **SAD on HRCT (n=9)** | **SAD/ILD on HRCT(n=8)** | **Normal (n=13)** | ***p*-value** |
| --- | --- | --- | --- | --- | --- |
| **Spirometry, L** |  |  |  |  |  |
| **FVC, % predicted** | 91.8 (88.5-104.2) | 80.2 (60.6-110.8) | 94.2 (83.9-107.4) | 102.3 (97.5-109.3) | 0.139 |
| **FEV1/FVC, % predicted** | 106.2 (101.0-108.9) | 96.3 (82.9-102.9) | 96..9 (94.1-103.1) | 103.1 (94.5-104.7) | 0.035 |
| **MMEF25/75, % predicted** | 63.8 (53.4-73.7) | 47.7 (41.1-63.2) | 60.1 (43.7-65.9) | 77.9 (72.4-99.5) | 0.007 |
| **RV, % predicted** | 92.5 (83.0-114.0) | 105.5 (87.0-111.5) | 117.5(102.0-134.0) | 106.0 (93.0-114.7) | 0.146 |
| **IOS, kPa/L/s** |  |  |  |  |  |
| **R5, % predicted** | 152.0 (131.0-165.0) | 195.0 (127.5-227.0) | 111.0 (98.5-165.0) | 110 (94.7-122.4) | 0.008 |
| **R20, % predicted** | 134.5 (115.0-154.0) | 132.0 (116.3-190.8) | 120.0 (101.0-175.0) | 116.0 (106.0-134.0) | 0.281 |
| **X5** | -0.18 (-0.22- -0.15) | -0.21 (-0.25- -0.12) | -0.15 (-0.27- -0.13) | -0.13(-0.15- -0.12) | 0.071 |
| **R5-R20** | 0.13 (0.05-0.22) | 0.16 (0.12-0.31) | 0.05 (0.02-0.13) | 0.05 (0.01-0.08) | 0.008 |
| **Frex** | 15.8 (13.1-24.2) | 19.6 (15.4-27.5) | 14.4 (12.1-22.1) | 12.1 (10.2-14.1) | 0.031 |
| **AX** | 0.99 (0.57-2.19) | 0.52 (0.35-1.56) | 1.70 (0.91-2.51) | 0.40 (0.25-0.59) | 0.005 |
| **DLCO, % predicted** | 69.9 (53.6-79.0) | 61.5 (49.6-72.2) | 62.0(45.3-79.7) | 71.3 (62.5-89.4) | 0.430 |

DLCO: diffusing capacity of the lung for carbon monoxide; FVC: forced vital capacity; FEV1: forced expiratory volume in one second; ILD: interstitial lung disease; SAD: small airway disease; HRCT: high-resolution computed tomography; IOS: impulse oscillometry; MMEF: maximal mid expiratory flow curve; RV: residual volume
